# Diagnostic implications of soluble triggering receptor expressed on myeloid cells-1 in patients with acute respiratory distress syndrome and abdominal diseases: a preliminary observational study

**DOI:** 10.1186/cc10015

**Published:** 2011-02-04

**Authors:** Paula Ramirez, Pedro Kot, Veronica Marti, Maria Dolores Gomez, Raquel Martinez, Vicente Saiz, Francisco Catala, Juan Bonastre, Rosario Menendez

**Affiliations:** 1 Department of Intensive Care Medicine, Hospital Universitario la Fe, Avda. Campanar 21, 46009 Valencia, Spain; 2 Department of Microbiology, Hospital Universitario la Fe, Avda. Campanar 21, 46009 Valencia, Spain; 3 Department of Pneumology, Hospital Universitario la Fe, Avda. Campanar 21, 46009 Valencia, Spain; 4 Department of Radiology, Hospital Universitario la Fe, Avda. Campanar 21, 46009 Valencia, Spain

## Abstract

**Introduction:**

Patients admitted to the intensive care unit (ICU) because of acute or decompensated chronic abdominal disease and acute respiratory failure need to have the potential infection diagnosed as well as its site (pulmonary or abdominal). For this purpose, we measured soluble triggering receptor expression on myeloid cells-1 (sTREM-1) in alveolar and peritoneal fluid.

**Methods:**

Consecutive patients (*n *= 21) with acute or decompensated chronic abdominal disease and acute respiratory failure were included. sTREM was measured in alveolar (A-sTREM) and peritoneal (P-sTREM) fluids.

**Results:**

An infection was diagnosed in all patients. Nine patients had a lung infection (without abdominal infection), 5 had an abdominal infection (without lung infection) and seven had both infections. A-sTREM was higher in the patients with pneumonia compared to those without pneumonia (1963 ng/ml (1010-3129) vs. 862 ng/ml (333-1011); *P *0.019). Patients with abdominal infection had an increase in the P-sTREM compared to patients without abdominal infection (1941 ng/ml (1088-3370) vs. 305 ng/ml (288-459); *P *< 0.001). A cut-off point of 900 pg/ml of A-sTREM-1 had a sensitivity of 81% and a specificity of 80% (NPV 57%; PPV 93%, AUC 0.775) for the diagnosis of pneumonia. In abdominal infections, a cut-off point for P-sTREM of 900 pg/ml had the best results (sensitivity 92%; specificity 100%; NPV 90%, PPV 100%, AUC = 0.903).

**Conclusions:**

sTREM-1 measured in alveolar and peritoneal fluids is useful in assessing pulmonary and peritoneal infection in critical-state patients-A-sTREM having the capacity to discriminate between a pulmonary and an extra-pulmonary infection in the context of acute respiratory failure.

## Introduction

Patients with acute or decompensated chronic abdominal diseases can develop acute respiratory insufficiency, the etiology of which is difficult to identify. The difficulty arises because the condition is a result of acute respiratory failure, which is caused by an inflammatory response that is secondary to the abdominal pathology or that is due to nosocomial pneumonia [1,2]. In this context, the diagnosis of an abdominal or lung infection can be complicated by several factors: (a) the systemic signs and symptoms of infection are non-specific, (b) the clinical data and the radiographic findings within the context of the patient in the intensive care unit (ICU) do not provide high specificity for either of the possibilities, and (c) the microbiological findings can be altered by previous antibiotic use. Hence, the therapeutic attitude, the management of the patient, and the prognosis would depend heavily on the identification of the focus of the infection.

The use of markers of systemic inflammation in the diagnosis and in therapeutic decision-making is progressively more valuable in clinical practice [3]. One of the more frequent applications is in the differential diagnosis between the inflammatory pictures of infection versus non-infection [4]. However, the measurement of inflammation markers in the circulation does not identify the focus of the infection [5]. Determinations of C-reactive protein or procalcitonin (PCT) in the alveolar fluid have been useless to diagnose infection as cytokines [6-8]. Conversely, the measurement of the triggering receptor expressed on myeloid cells 1 (TREM-1) in alveolar, pleural, sinovial, and cerebrospinal fluids has, indeed, been demonstrated to be useful in several studies [5,9-12]. Also, an increase in TREM-1 has been observed in peritoneal fluid following the induction of peritonitis in an animal model [13].

Our hypothesis for this study is that the determination of soluble TREM-1 (sTREM-1) in alveolar and peritoneal fluids in seriously ill patients with abdominal diseases and respiratory insufficiency could be useful in identifying the existence of an infection. It is plausible that the local increase in sTREM-1 would be higher in the presence of infection, and this would enable us to distinguish pulmonary or extrapulmonary infection as the etiology of acute respiratory failure.

The objective of the present study was to investigate the diagnostic value of sTREM in bronchoalveolar lavage and peritoneal fluid in patients admitted to the ICU with severe respiratory insufficiency and an abdominal disease. We wished, as a secondary objective, to compare the diagnostic value of cutoff points of sTREM in both of these biological fluids.

## Materials and methods

### Design of the study

We conducted a prospective observation study of consecutive cases.

### Study site and subjects

The study was conducted in the ICU for a period of 18 months. The patients selected needed to fulfill the following criteria: (a) acute abdominal pathology, (b) respiratory insufficiency with acute respiratory distress syndrome (ARDS) criteria of not more than 3 days in duration, and (c) admission to the ICU. We excluded patients in whom it was not possible to extract a sample of peritoneal fluid. The protocol was reviewed and approved by the local ethics committee, and the patients (or their relatives) provided informed consent to participation in the study. The written consent included the permission to collect and publish (anonymously) personal data concerning the patients.

### Protocol for data collection

The following data were collected: age, gender, chronic diseases, vital signs, Acute Physiology Score, Acute Physiology and Chronic Health Evaluation II (APACHE II) score [14], Sepsis-related Organ Failure Assessment (SOFA) score [15], presence or absence of systemic inflammatory response syndrome [16], data on gas exchange and the mode of mechanical ventilation, radiological assessments, and the score on the modified Clinical Pulmonary Infection Score (CPIS) [17]. With respect to the abdomen, data were collected via physical examination, and the intra-abdominal pressure was measured via vesical probe. Other data included radiological assessments, intraoperative findings, blood chemistry, and microbiology laboratory findings. With respect to the lung, data were collected on the macroscopic aspects of the respiratory secretions, the Gram bacteria staining of mini-bronchoalveolar lavage (mini-BAL) fluid sent to the microbiology laboratory, and the quantitative isolations in culture.

### Definitions

Diagnosis of hospital-acquired pneumonia, the pneumonia associated with mechanical ventilation, or pneumonia related to the health-care provision was conducted in accordance with the criteria recommended by the American Thoracic Society and the Infectious Diseases Society of America [18]. The diagnosis of the abdominal infection focus was performed in accordance with the Centers for Disease Control and Prevention (Atlanta, GA, USA) criteria for gastrointestinal infection and for infections associated with surgery [19].

### Collection and processing of the isolated abdominal fluid

Fine-needle aspiration puncture was performed under echographic guidance by experienced interventional radiologists. After vortex mixing, the sample was separated into three aliquots: the first was stored at -70°C until required for analysis, the second was sent for cytobiochemical analyses, and the third was sent to the microbiology laboratory.

### Collection and processing of the alveolar liquid

The sample of alveolar liquid was obtained using a small (20 mL of physiologic saline) volume of bronchoalveolar lavage (mini-BAL) [20]. After vortex mixing, the sample was centrifuged into two phases: the supernatant was separated and frozen at -70°C until required for analyses, and the infranatant was sent to the microbiology laboratory. The cutoff value of mini-BAL for the diagnosis of lung infection was 10^3 ^colony-forming units per milliliter.

### Measurement of inflammation markers

Serum PCT was measured with time-resolved amplified cryptate emission (TRACE) technology in a Kryptor analyzer (Brahms Diagnostica, Berlin, Germany). The sTREM-1 was determined by immunoassay with a combination monoclonal/polyclonal antibody of the IgG1 type raised against TREM-1 (R&D Systems, Inc., Minneapolis, MN, USA). The assay was performed in accordance with the instructions of the manufacturer.

### Statistical analyses

All statistical analyses were performed with SPSS version 15 software (SPSS, Inc., Chicago, IL, USA). The χ^2 ^test was used for categorical variables, and the Student *t *or Mann-Whitney test was used for continuous variables. The values for PCT and sTREM-1 were expressed as medians with the interquartile ranges (25% to 75%) in parenthesis. Diagnostic capacities of alveolar sTREM-1, peritoneal sTREM-1, and the alveolar-to-peritoneal sTREM-1 ratios were evaluated with the receiver operating characteristic curves. Sensitivity and specificity as well as positive (PPV) and negative (NPV) predictive values were calculated.

## Results

Twenty-two patients fulfilled the inclusion criteria. One patient was censored because of our inability to obtain abdominal fluid. The mean age (± standard deviation) was 48.2 ± 16.7 years, and 57% (*n *= 12) were males. Eleven patients (52%) had a chronic abdominal disease, seven patients (33%) had hepatic cirrhosis, and one patient each had intestinal graft-versus-host disease, Budd-Chiari syndrome, cystic fibrosis, and intestinal lymphoma. The acute abdominal diseases diagnosed were spontaneous bacterial peritonitis in 29% of cases, acute enteritis in 19%, acute pancreatitis in 14%, digestive tract hemorrhage in 14%, and acute hepatitis in 10%, and 1 case each (5%) had cholecystitis, hepatic abscess, and intestinal subocclusion. The mean score on the APACHE II scale on the day of admission to the ICU was 18.6 ± 5.8 points. The intra-ICU mortality was 76.2%. Table 1 shows the individual characteristics of the patients in the study.

**Table 1:** Baseline characteristics of the patients

Case	Chronic abdominal pathology	Other complaints	Admission to hospital	APACHE II score	Exitus
1	No	No	Hepatic lesions	13	Yes
3	Intestinal GVHD	Acute myeloid leukemia	Sepsis	15	Yes
4	Hepatic cirrhosis	No	Gastrointestinal bleeding	24	Yes
5	No	Acute lymphatic leukemia	Enteritis	19	
6	No	No	Acute pancreatitis	18	No
7	Hepatic cirrhosis	AIDS	Gastrointestinal bleeding	21	Yes
8	Hepatic cirrhosis	No	Gastrointestinal bleeding	20	No
9	Budd-Chiari syndrome	No	Respiratory failure	10	Yes
10	No	No	Acute pancreatitis	20	Yes
11	No	No	Intestinal subocclusion	25	No
12	No	Alcoholism	Acute pancreatitis	23	Yes
13	No	AIDS	Acute hepatitis	12	Yes
14	No	No	Paralyzed ileum		Yes
15	Hepatic cirrhosis	AIDS	Hydropic decompensation	29	Yes
16	No	Acute myeloid leukemia	Sepsis	19	Yes
17	Cystic fibrosis	Hepato-bipulmonary transplant	Hepato-bipulmonary transplant	9	Yes
18	Hepatic cirrhosis	No	Cholecystitis	11	No
19	Intestinal lymphoma	No	Acute hepatitis	14	No
20	No	No	Acute pancreatitis	25	Yes
21	Hepatic cirrhosis	No	Gastrointestinal bleeding	19	Yes
22	Hepatic cirrhosis	No	Hydropic decompensation	26	Yes

Patient 2 was removed from the analyses because of our inability to aspirate peritoneal fluid. APACHE II, Acute Physiology and Chronic Health Evaluation II; GVHD, graft-versus-host disease.

### General characteristics upon entry into the study

The mean stay in the ICU was 4.04 ± 2.3 days, and the mean duration of ventilation was 2.85 ± 1.2 days. The mean body temperature was 38.3 ± 1°C, the leukocytes were 10,176 ± 6,736 cells/mL (median 11,600, range 4,600 to 13,650), and the plasma PCT was 17.77 ± 25.42 ng/mL (median 7.9, range 1.84 to 18.96). The mean SOFA score was 12.8 ± 3.4 points. All of the cases required wide-spectrum antibiotic treatment and invasive mechanical ventilation with an elevated fraction of inspired oxygen (FiO_2 _= 0.7 ± 0.2 and positive end-expiratory pressure = 9 ± 2.5 mm Hg). Fourteen patients (66.7%) were in shock with a need for vasoactive drugs, and four (19%) underwent the technique of continuous renal replacement therapy (Table 2).

**Table 2:** Characteristics of the patients upon inclusion in the study

Case	SOFA score	Antibiotics	Vasoactive drugs	CRRT	Procalcitonin, ng/mL	Temperature, °C	Leukocytes,/mm^3^	Final diagnosis
1	11	Yes	Yes	No	96.46	39.0	9,800	Systemic infection^a^
3	14	Yes	Yes	No	40.9	37.0	1,100	Systemic infection^b^
4	12	Yes	No	No	1.28	36.4	7,600	VAP
5	16	Yes	No	No	18.62	40.0	0	Enteritis + VAP
6	12	Yes	Yes	Yes	15.0	38.6	21,200	VAP + infected pancreatitis
7	13	Yes	Yes	No	3,054	38.8	11,700	Nosocomial pneumonia
8	16	Yes	Yes	No	3.5	39.0	2,900	VAP
9	14	Yes	Yes	No	10.38	28.0	20,400	HAP
10	15	Yes	Yes	Yes	50.0	38.0	11,600	VAP + infected pancreatitis
11	9	Yes	Yes	No	4.53	37.8	14,100	VAP
12	11	Yes	No	Yes	13.0	38.2	7,600	Infected pancreatitis
13	11	Yes	No	No	0.833	38.0	12,700	SBP
14	11	Yes	Yes	No	68.65	40.0	13,200	Enteritis
15	21	Yes	Yes	No	19.3	37.6	12,400	SBP
16	16	Yes	Yes	No	7.9	39.3	200	Enteritis
17	6	Yes	No	No	0.784	36.0	6,600	Systemic infection^c^
18	7	Yes	No	No	0.49	38.8	11,900	HAP
19	16	Yes	Yes	No	0.90	37.0	6,100	HAP
20	12	Yes	Yes	Yes	10.74	38.8	23,200	VAP
21	12	Yes	No	No	2.41	38.7	3,100	VAP
22	14	Yes	Yes	No	4.44	38.0	16,300	VAP + SBP

Patient 2 was removed from the analyses because of our inability to aspirate peritoneal fluid. 'Systemic infection' indicates that the same infection affected both the abdomen and lungs: ^a^septic thrombophlebitis of the portal vein by *Salmonella tiphy *with hematogenous pneumonia; ^b^citomegaloviruses colitis and pneumonia; ^c^systemic infection (lung + abdominal) by *Aspergillus fumigates*. CRRT, continuous renal replacement therapy; HAP, hospital-acquired pneumonia; SBP, spontaneous bacterial peritonitis; SOFA, Sepsis-related Organ Failure Assessment; VAP, ventilator-associated pneumonia.

### Respiratory characteristics at the time of inclusion in the study

The mean score on the CPIS was 5.4 ± 2.4 points (median 6, range 3 to 7). The mean parameters of gas exchange were pH = 7.33 ± 0.11, partial pressure of oxygen (pO_2_) = 42.2 ± 12.7 mm Hg, partial pressure of carbon dioxide (pCO_2_) = 82.9 ± 28.3 mm Hg, bicarbonate = 19.9 ± 3.3 mmol/L, and arterial partial pressure of oxygen (PaO_2_)/FiO_2 _ratio = 122.7 ± 43.4. Radiological findings were 8 localized condensations (38%), 11 diffuse interstitial infiltrate (52.5%), and 2 pleural effusions (9.5%). Sixteen patients (76%) had a definitive diagnosis of pulmonary infection: 7 of them also had an abdominal infection (in 5 of these, the infection was systemic and caused by the same microorganism). The median alveolar sTREM-1 was 1,437 (range 656 to 2,512) pg/mL (Table 3).

**Table 3:** Respiratory characteristics of the patients

Case	Days under invasive MV	PaO_2_/FiO_2_	Chest x-ray	CPIS	Alveolar microbiology	Alveolar sTREM-1, pg/mL	Lung infection	Type of infection
1	3	167	Diffused interstitial	3	*Salmonella tiphy*	1,437	Yes	HAP
3	1	111	Diffused interstitial	3	CMV	434	Yes	HAP
4	2	83	Diffused interstitial	6	*Acinetobacter baumannii*	2,475	Yes	HAP
5	7	69	LRL condensation	8	*A. baumannii*	430	Yes	VAP
6	6	180	LLL condensation	6	*A. baumannii*	2,166	Yes	VAP
7	4	80	Bilateral infiltrate	6	*Escherichia coli*	1,755	Yes	HAP
8	2	172	Diffused interstitial	5	*Staphylococcus aureus*	3,322	Yes	HAP
9	2	111	Pulmonary condensation, R	7	*Aspergillus fumigatus*	3,399	Yes	HAP
10	3	108	Bilateral infiltrate	6	*Haemophilus influenzae*	3,758	Yes	VAP
11	4	190	LRL condensation	6	*E. coli*	1,167	Yes	HAP
12	2	93	Bilateral infiltrate	1	Negative	862	No	
13	3	132	Pleural effusion, R	2	Negative	229	No	
14	1	160	Diffused interstitial	4	Negative	883	No	
15	1	91	Diffused interstitial	4	*Candida albicans*	1,139	No	
16	1	64	Diffused interstitial	4	Negative	437	No	
17	1	195	Diffused interstitial	3	*A. fumigatus*	2,382	Yes	HAP
18	1	125	Bilateral pleural effusion	9	*Pseudomonas aeruginosa*	175	Yes	HAP
19	2	130	Bi-basal condensation	5	*A. fumigatus*	2,550	Yes	HAP
20	6	58	Pulmonary condensation, L	8	*P. aeruginosa*	958	Yes	VAP
21	6	95	LRL condensation	7	*A. baumannii*	450	Yes	VAP
22	2	163	LRL condensation	8	*S. aureus*	3,986	Yes	HAP

Patient 2 was removed from the analyses because of our inability to aspirate peritoneal fluid. CPIS, Clinical Pulmonary Infection Score; HAP, hospital-acquired pneumonia; L, left; LLL, lower left lobe; LRL, lower right lobe; MV, mechanical ventilation; PaO_2_/FiO_2_, arterial partial pressure of oxygen/fraction of inspired oxygen; R, right; VAP, ventilator-associated pneumonia.

### Abdominal characteristics on the day of inclusion in the study

The mean level of glucose in the peritoneal fluid was 157.64 ± 77 mg/dL (median 161, range 104 to 330), and the mean of neutrophils was 406.5 ± 1,108 cells/mm^3 ^(median 51, range 10 to 249). The mean intra-abdominal pressure was 15.06 mm Hg. The diagnosis of abdominal infection was established in 12 patients (57%); in 7 of these patients, the diagnosis of lung infection was established as well. The median value of s-TREM in peritoneal fluid was 933 (range 305 to 2,560) pg/mL (Table 4).

**Table 4:** Abdominal characteristics of the patients

Case	IAP, mm Hg	Neutrophils in peritoneal fluid, mm^3^	Glucose in peritoneal fluid, mg/dL	Peritoneal fluid microbiology	Peritoneal sTREM-1, pg/mL	Abdominal infection	Type of infection
1		130		*Salmonella tiphy*	305	Yes	Hepatic abscesses
3	15	1,670	228	Polymicrobial	2,871	Yes	Colitis CMV
4	18	0	179	Negative	482	No	
5	18			*Acinetobacter baumannii*	935	Yes	Enteritis
6		103	154	*A. baumannii*	1,242	Yes	Pancreatic infection
7	18	10	238	Negative	445	No	
8		10	128	Negative	288	No	
9	13	30	45	Negative	459	No	
10	22	12,700		Negative	3,474	Yes	Pancreatic infection
11		16	248	Negative	227	No	
12	15	0	127	*Enterococcus faecalis*	3,267	Yes	Pancreatic infection
13	11	544	93	Negative	1,423	Yes	SBP
14	11			Negative	2,250	Yes	Enteritis^a^
15	7	462	34	Negative	1,633	Yes	SBP
16	17	0	186	Negative	933	Yes	Enteritis^b^
17				*Candida krusei*, *Enterococcus faecium*	3,634	Yes	Enteritis^c^
18	11	51	169	Negative	305	No	
19	25	249	108	Negative	301	No	
20	14	10		Negative	854	No	
21	14	148	285	Negative	174	No	
22	12	4,365	30	*Escherichia coli*	4,406	Yes	SBP

Patient 2 was removed from the analyses because of our inability to aspirate peritoneal fluid. ^a^Diagnosed from surgical findings; ^b^diagnosed from necropsy findings; ^c^clinical and microbiological diagnoses. CMV, cytomegalovirus; IAP, intra-abdominal pressure; SBP, spontaneous bacterial peritonitis; sTREM-1, soluble triggering receptor expressed on myeloid cells 1.

### Capacities of A-sTREM and P-sTREM to diagnose lung and abdominal infections, respectively

Nine patients had lung infection (without abdominal infection), 5 had abdominal infection (without lung infection), and 7 had both infections. The patients with lung infection had a higher CPIS and a greater alveolar sTREM-1 (*P *= 0.019 and *P *= 0.019, respectively) compared with those without lung infection. The patients with abdominal infection had a lower CPIS and increased plasma PCT and peritoneal sTREM (*P *= 0.002, *P *= 0.018, *P *< 0.001, respectively) compared with those without abdominal infection (Tables 5 and 6).

**Table 5:** Identification of pulmonary (alveolar) and abdominal (peritoneal) infection

	Lung infection			Abdominal infection		
		
	Yes	No	*P *value	Yes	No	*P *value
CPIS	6.5 (3.7-7.7)	4 (1.5-4.5)	0.019	3.5 (3.5-5.7)	7 (6-8)	0.002
Serum PCT, ng/mL	4.5 (1.9-16.8)	13 (7.9-19.3)	0.409	16.8 (6.2-45.4)	3.05 (6-8)	0.018
A-sTREM, pg/mL	1,963 (1,010-3,129	862 (333-1,011)	0.019	1,011 (435-2,274)	1,760 (1,167-2,550)	0.177
P-sTREM, pg/mL	470 (303-2056)	1,633 (1,423-2,250)	0.117	1,941 (1,088-3,370)	305 (288-459)	<0.001

A-sTREM, alveolar soluble triggering receptor expressed on myeloid cells; CPIS, Clinical Pulmonary Infection Score; PCT, procalcitonin; P-sTREM, peritoneal soluble triggering receptor expressed on myeloid cells.

**Table 6:** Diagnostic capacity of alveolar sTREM and peritoneal sTREM

	Abdominal infection	Lung infection
	Peritoneal sTREM ≥900 pg/mL	Alveolar sTREM ≥900 pg/mL
Sensitivity	92%	81%
Specificity	100%	80%
Positive predictive value	100%	93%
Negative predictive value	90%	57%
Area under the curve	0.903 (0.078)	0.775 (0.124)

sTREM, soluble triggering receptor expressed on myeloid cells.

The best cutoff point of alveolar sTREM for the diagnosis of lung infection was 900 pg/mL (sensitivity 81%, specificity 80%, PPV 93%, NPV 57%, and area under the curve [AUC] 0.775). In abdominal infection, the best cutoff point of peritoneal sTREM was 900 pg/mL (sensitivity 92%, specificity 100%, PPV 100%, NPV 90%, and AUC 0.903).

### Diagnostic capacity of the alveolar-to-peritoneal sTREM ratio to discriminate the infection focus

Nine patients had lung infection (without abdominal infection), 5 had abdominal infection (without lung infection), and 7 had both infections. All patients with just lung infection had an alveolar-to-peritoneal sTREM ratio of greater than 1, and all patients with just abdominal infection had an alveolar-to-peritoneal sTREM ratio of less than 1. However, patients with both infections had a huge variability, preempting any effective clinical application of the ratio.

## Discussion

The results of our study demonstrate the usefulness (high predictive value) of measuring sTREM-1 in alveolar and peritoneal fluids in the diagnosis of pulmonary or abdominal infection (or both) in the context of ARDS. A-sTREM-1 was able to identify pneumonia as a pathogenic factor for ARDS. The relationships between the alveolar and peritoneal sTREM-1 values identified the focus of the infection.

The application of the sTREM-1 measurement for diagnosing pulmonary infections has had conflicting results. In the original study by Gibot and colleagues [5] and in subsequent studies [10], the measurement of alveolar sTREM achieved good results. Gibot and colleagues [5] found an area under the receiver operating characteristic curve for alveolar sTREM-1 of 0.93 (95% confidence interval 0.92 to 0.95) in patients with community-acquired pneumonia or ventilator-associated pneumonia (VAP). In their study, Determann and colleagues [10] established a cutoff of 200 pg/mL of alveolar sTREM-1 with a sensibility of 75% and a specificity of 84% in the diagnosis of VAP. More recent studies by Anand and colleagues [21] and by others [22] did not reach the same conclusions. The discordance in the findings could be due to differences in the techniques for alveolar sample acquisition, in the method of measurement of sTREM-1, or in the type of patients included in the study. Anand and colleagues [21] segregated their patient population as those without VAP (*n *= 21), with definite VAP (*n *= 19), with indefinite VAP (*n *= 56), and with alveolar hemorrhage (*n *= 9) and analyzed only the first two of these groups. Although the group with VAP showed higher levels of sTREM-1 (171.9 ± 158.7 pg/mL) than the group without VAP (96.7 ± 76.2 pg/mL), this difference did not reach statistical significance (*P *= 0.06) [21]. In our study, the patients with lung infection had a higher level of alveolar sTREM than the patients without lung infection (mean 1,963 pg/mL, interquartile range 1,010 to 3,129 versus 862 pg/mL, interquartile range 333 to 1,011; *P *= 0.019). Of note is that the values of sTREM-1 observed in our study do not compare with those observed by Anand and colleagues [21], who used the same analytical method as we did (that is, enzyme-linked immunoabsorbent assay). The differences could be due to the extreme status of our patient population (SOFA score 12.8 ± 3.4); the study of Anand and colleagues does not report SOFA score. With a cutoff point of 900 pg/mL, the specificity is high and the PPV reaches 100%.

The measurement of sTREM-1 in peritoneal fluid as a diagnostic method has been less studied. It has been tested in an animal model in which the induction of peritonitis provoked an increase in the sTREM-1 in peritoneal fluid [13]. Recently, Determann and colleagues [23] analyzed the capacity of peritoneal sTREM-1 to diagnose the persistence of secondary peritonitis post-surgery. The authors, in a sequential study of sTREM-1, observed that the patients with persistent infection at 48 hours post-surgery had a significantly higher median sTREM-1 (319 versus 85 pg/mL; *P *= 0.001). We confirmed that patients with abdominal infection had elevated levels of peritoneal sTREM-1 of 1,941 pg/mL (interquartile range 1,088 to 3,370) versus 305 pg/mL (interquartile range 288 to 459) (*P *< 0.001). Furthermore, with a cutoff point of at least 900 pg/mL, the diagnostic value showed high sensitivity (92%), specificity (100%), PPV (100%), and NPV (90%).

As expected, body temperature and plasma leukocyte counts were ineffective in identifying the infection focus. Elevated levels of plasma PCT were associated with abdominal infection, whereas 60% of the patients with pulmonary infection had a serum PCT level of less than 2.5 ng/mL.

In our study, we used the measurement of sTREM-1 in alveolar and peritoneal fluids to discriminate the etiology of acute respiratory failure. However, the relatively high percentage of patients who have a systemic infection coexisting with abdominal and pulmonary infections complicates this objective. All patients with lung infection alone had an alveolar-to-peritoneal sTREM ratio of greater than 1, and all patients with abdominal infection alone had an alveolar-to-peritoneal sTREM ratio of less than 1. However, patients with both infections had a huge variability, preempting an effective clinical application of the ratio.

The principal limitation of our study is the small sample size. This important limitation, which precludes the generalization of the findings, is partially balanced by the novelty of the two aspects of the study design (that is, the application of sTREM-1 to the diagnosis of abdominal infection and the concomitant determination of the sTREM-1 in two different sites to establish the infection focus). Our results need to be corroborated in a study with a larger sample size. The second limitation is the heterogeneity of our cohort. We included neutropenic patients in whom the usefulness of sTREM-1 has not been established. However, in our neutropenic patients, peritoneal and alveolar sTREM-1 levels showed results similar to those in non-neutropenic patients. Although the diagnosis of infection had been performed in accordance with established criteria, the microbiology results could have been affected by the generalized use of broad-spectrum antibiotics.

## Conclusions

The results of our study show that the measurement of sTREM-1 is useful in the diagnosis of pulmonary infection and of abdominal infection in the context of severe acute respiratory failure. Further studies with a larger sample sizes are fully warranted to confirm the usefulness of sTREM-1 found in this preliminary study. Moreover, on the basis of our findings, the accuracy of this marker in neutropenic patients should be explored.

## Key messages

• Alveolar soluble triggering receptor expressed on myeloid cells 1 (sTREM-1) is useful in diagnosing lung infections in the context of acute respiratory distress syndrome.

• Peritoneal sTREM-1 is capable of identifying an abdominal infection, including those developed in the setting of a chronic abdominal disease as spontaneous bacterial peritonitis in patients with hepatic cirrhosis.

• sTREM-1 seems to be the ideal biomarker to identify the site of infection in critical care patients when measured in fluids coming from the suspected tissues.

## Abbreviations

APACHE II: Acute Physiology and Chronic Health Evaluation II; ARDS: acute respiratory distress syndrome; AUC: area under the curve; CPIS: Clinical Pulmonary Infection Score; FiO_2_: fraction of inspired oxygen; ICU: intensive care unit; mini-BAL: mini-bronchoalveolar lavage; NPV: negative predictive value; PCT: procalcitonin; PPV: positive predictive value; SOFA: Sepsis-related Organ Failure Assessment; sTREM-1: soluble triggering receptor expressed on myeloid cells 1; TREM-1: triggering receptor expressed on myeloid cells 1; VAP: ventilator-associated pneumonia.

## Competing interests

The authors declare that they have no competing interests.

## Authors' contributions

PR, JB, and RMe contributed to the design of the study, analysis of the data, and manuscript preparation. PK and VM contributed to patient recruitment and manuscript preparation. MDG contributed to analysis of biomarkers (sTREM). RMa contributed to patient recruitment and sample aspiration. VS and FC contributed to patient recruitment and peritoneal liquid aspiration. All authors read and approved the final manuscript.
